# LIVING WITH END-STAGE LIVER DISEASE: PERSPECTIVES OF YOUNGER AND OLDER PATIENT–CARE PARTNER DYADS

**DOI:** 10.1093/geroni/igac059.2653

**Published:** 2022-12-20

**Authors:** Lissi Hansen, Susan Rosenkranz, Shirin Hiatt

**Affiliations:** Oregon Health & Science University, Portland, Oregon, United States; Oregon Health & Science University, Portland, Oregon, United States; Oregon Health & Science University, Portland, Oregon, United States

## Abstract

The uncertain trajectory of end-stage liver disease (ESLD) leaves patients and their informal care-partners to face psychological, physical, social, and financial burdens, complicating their relationship. Knowledge is lacking on how living with ESLD affects patients and informal care-partners. The study purpose was to compare how ESLD affects relationships between younger and older patient-care-partner dyads. Patients with ESLD and informal care-partners were recruited through liver clinics at two hospital settings. They completed questionnaires including two open-ended questions about how ESLD affected their relationship (R01NR016017, NINR/NIH). Patient eligibility criteria: age >21 and MELD-Na score >15. Care-partner eligibility criteria: age ≥18 and identified as patient’s primary support. Conventional content and qualitative dyadic analysis were used to analyze written responses to the two questions. Data were available for 140 patient-care-partner dyads. Patients averaged 57 years old (range, 23-83 years; 69% were male; 31% with ETOH as primary etiology). Care partners averaged 57.5 years old (range, 19-85 years; 74% were female). Older dyads were more likely to report having a closer relationship, although tempered by uncertainty and symptoms limiting activities. Younger dyads emphasized managing symptoms, financial stress, caregiving burden, and lack of intimacy among spousal/partners. Diverging and overlapping themes within dyads reflected caregiving challenges, relationship strain, and support from other relationships. Clinicians should provide information and identify helpful resources that support the patient- care-partner dyad and their ability to manage ESLD. Future longitudinal dyadic studies should examine the patient-care-partner relationship at different points in the disease progression to develop interventions that help living with ESLD.

